# Supplementary material for: A Critical View of the Application of the APEX Software (Aqueous Photochemistry of Environmentally-Occurring Xenobiotics) to Predict Photoreaction Kinetics in Surface Freshwaters
Source: Molecules. 2019 Dec 18;25(1):9. doi: 10.3390/molecules25010009 (PMC7017383; doi:10.3390/molecules25010009)
Supplement: Supplementary file 1 [file molecules-25-00009-s001.zip › 3.2.4_gcc-4.4.0.zip!/3.2.4_gcc-4.4.0/3.2.4_gcc-4.4.0/share/octave/packages/control-1.0.11/doc/control.pdf]

Octave Control Systems Toolbox (OCST)

Version 1.0.0
July 2008

Dr A Scottedward Hodel

Copyright © 2008 A Scottedward Hodel

Permission is granted to make and distribute verbatim copies of this manual provided the copyright notice and this permission notice are preserved on all copies.

Permission is granted to copy and distribute modified versions of this manual under the conditions for verbatim copying, provided that the entire resulting derived work is distributed under the terms of a permission notice identical to this one.

Permission is granted to copy and distribute translations of this manual into another language, under the same conditions as for modified versions.

Table of Contents

1 Introduction 1

2 System Data Structure 2
- 2.1 Variables common to all OCST system formats 2
- 2.2 tf format variables 3
- 2.3 zp format variables 3
- 2.4 ss format variables 3

3 System Construction and Interface Functions 4
- 3.1 Finite impulse response system interface functions 4
- 3.2 State space system interface functions 5
- 3.3 Transfer function system interface functions 10
- 3.4 Zero-pole system interface functions 11
- 3.5 Data structure access functions 13

4 System display functions 18

5 Block Diagram Manipulations 19

6 Numerical Functions 27

7 System Analysis-Properties 32

8 System Analysis-Time Domain 37

9 System Analysis-Frequency Domain 41

10 Controller Design 45

11 Miscellaneous Functions (Not yet properly filed/documented) 53

1 Introduction

The Octave Control Systems Toolbox (OCST) was initially developed by Dr. A. Scottedward Hodel a.s.hodel@eng.auburn.edu with the assistance of his students

- R. Bruce Tenison btenison@dibbs.net,
- David C. Clem,
- John E. Ingram John.Ingram@sea.siemans.com, and
- Kristi McGowan.

This development was supported in part by NASA's Marshall Space Flight Center as part of an in-house CACSD environment. Additional important contributions were made by Dr. Kai Mueller mueller@ifr.ing.tu-bs.de and Jose Daniel Munoz Frias (`place.m`).

An on-line menu-driven tutorial is available via `DEMOcontrol`; beginning OCST users should start with this program.

DEMOcontrol Function File

Octave Control Systems Toolbox demo/tutorial program. The demo allows the user to select among several categories of OCST function:

```
octave:1> DEMOcontrol
Octave Controls System Toolbox Demo

[ 1] System representation
[ 2] Block diagram manipulations
[ 3] Frequency response functions
[ 4] State space analysis functions
[ 5] Root locus functions
[ 6] LQG/H2/Hinfinity functions
[ 7] End
```

Command examples are interactively run for users to observe the use of OCST functions
See also: bddemo, frdemo, analdemo, moddmeo, rldemo

2 System Data Structure

The OCST stores all dynamic systems in a single data structure format that can represent continuous systems, discrete-systems, and mixed (hybrid) systems in state-space form, and can also represent purely continuous/discrete systems in either transfer function or pole-zero form. In order to provide more flexibility in treatment of discrete/hybrid systems, the OCST also keeps a record of which system outputs are sampled.

Octave structures are accessed with a syntax much like that used by the C programming language. For consistency in use of the data structure used in the OCST, it is recommended that the system structure access m-files be used (see Chapter 3 [sysinterface], page 4). Some elements of the data structure are absent depending on the internal system representation(s) used. More than one system representation can be used for SISO systems; the OCST m-files ensure that all representations used are consistent with one another.

sysrepdemo Function File

Tutorial for the use of the system data structure functions

2.1 Variables common to all OCST system formats

The data structure elements (and variable types) common to all system representations are listed below; examples of the initialization and use of the system data structures are given in subsequent sections and in the online demo `DEMOcontrol`.

n
nz The respective number of continuous and discrete states in the system (scalar)

inname
outname list of name(s) of the system input, output signal(s). (list of strings)

sys System status vector. (vector)

This vector indicates both what representation was used to initialize the system data structure (called the primary system type) and which other representations are currently up-to-date with the primary system type (see Section 3.5 [structaccess], page 13).

The value of the first element of the vector indicates the primary system type.

0 for tf form (initialized with `tf2sys` or `fir2sys`)

1 for zp form (initialized with `zp2sys`)

2 for ss form (initialized with `ss2sys`)

The next three elements are boolean flags that indicate whether tf, zp, or ss, respectively, are “up to date" (whether it is safe to use the variables associated with these representations). These flags are changed when calls are made to the `sysupdate` command.

tsam Discrete time sampling period (nonnegative scalar). *tsam* is set to 0 for continuous time systems.

yd Discrete-time output list (vector)

indicates which outputs are discrete time (i.e., produced by D/A converters) and which are continuous time. yd(ii) = 0 if output ii is continuous, = 1 if discrete.

The remaining variables of the system data structure are only present if the corresponding entry of the `sys` vector is true (=1).

2.2 `tf` format variables

num numerator coefficients (vector)

den denominator coefficients (vector)

2.3 `zp` format variables

zer system zeros (vector)

pol system poles (vector)

k leading coefficient (scalar)

2.4 `ss` format variables

a
b
c
d The usual state-space matrices. If a system has both continuous and discrete states, they are sorted so that continuous states come first, then discrete states

Note some functions (e.g., `bode`, `hinfsyn`) will not accept systems with both discrete and continuous states/outputs

stname names of system states (list of strings)

3 System Construction and Interface Functions

Construction and manipulations of the OCST system data structure (see Chapter 2 [sysstruct], page 2) requires attention to many details in order to ensure that data structure contents remain consistent. Users are strongly encouraged to use the system interface functions in this section. Functions for the formatted display in of system data structures are given in Chapter 4 [sysdisp], page 18.

3.1 Finite impulse response system interface functions

fir2sys (*num*, *tsam*, *inname*, *outname*) Function File

construct a system data structure from FIR description

Inputs

num vector of coefficients $[c_0, c_1, \ldots, c_n]$ of the SISO FIR transfer function

$$C(z) = c_0 + c_1 z^{-1} + c_2 z^{-2} + \ldots + c_n z^{-n}$$

tsam sampling time (default: 1)

inname name of input signal; may be a string or a list with a single entry

outname name of output signal; may be a string or a list with a single entry

Output

sys system data structure

Example

```
octave:1> sys = fir2sys([1 -1 2 4],0.342,\
> "A/D input","filter output");
octave:2> sysout(sys)
Input(s)
1: A/D input

Output(s):
1: filter output (discrete)

Sampling interval: 0.342
transfer function form:
1*z^3 - 1*z^2 + 2*z^1 + 4
-------------------------
1*z^3 + 0*z^2 + 0*z^1 + 0
```

[*c*, *tsam*, *input*, *output*] = **sys2fir** (*sys*) Function File

Extract FIR data from system data structure; see `fir2sys` for parameter descriptions

See also: fir2sys

3.2 State space system interface functions

outsys = **ss** (*a*, *b*, *c*, *d*, *tsam*, *n*, *nz*, *stname*, *inname*, *outname*, *outlist*) — Function File

Create system structure from state-space data. May be continuous, discrete, or mixed (sampled data)

Inputs

a
b
c
d — usual state space matrices

default: d = zero matrix

tsam — sampling rate. Default: $tsam = 0$ (continuous system)

n
nz — number of continuous, discrete states in the system

If *tsam* is 0, $n =$ `rows`(a), $nz = 0$

If *tsam* is greater than zero, $n = 0$, $nz =$ `rows`(a)

see below for system partitioning

stname — cell array of strings of state signal names

default (*stname*=[] on input): `x_n` for continuous states, `xd_n` for discrete states

inname — cell array of strings of input signal names

default (*inname* = [] on input): `u_n`

outname — cell array of strings of output signal names

default (*outname* = [] on input): `y_n`

outlist

list of indices of outputs y that are sampled

If *tsam* is 0, *outlist* = []

If *tsam* is greater than 0, $outlist = 1 :$ `rows`(c)

Unlike states, discrete/continuous outputs may appear in any order

`sys2ss` returns a vector *yd* where $yd(outlist) = 1$; all other entries of *yd* are 0

Output

outsys — system data structure

System partitioning

Suppose for simplicity that outlist specified that the first several outputs were continuous and the remaining outputs were discrete. Then the system is partitioned as

```
x = [ xc ]  (n x 1)
[ xd ]  (nz x 1 discrete states)
a = [ acc acd ]  b = [ bc ]
[ adc add ]      [ bd ]
c = [ ccc ccd ]  d = [ dc ]
[ cdc cdd ]      [ dd ]

(cdc = c(outlist,1:n), etc.)
```

with dynamic equations:

$$\frac{d}{dt}x_c(t) = a_{cc}x_c(t) + a_{cd}x_d(k * t_{sam}) + bc * u(t)$$

$$x_d((k+1) * t_{sam}) = a_{dc}x_c(kt_{sam}) + a_{dd}x_d(kt_{sam}) + b_d u(kt_{sam})$$

$$y_c(t) = c_{cc}x_c(t) + c_{cd}x_d(kt_{sam}) + d_c u(t)$$

$$y_d(kt_{sam}) = c_{dc}x_c(kt_{sam}) + c_{dd}x_d(kt_{sam}) + d_d u(kt_{sam})$$

Signal partitions

```
| continuous      | discrete               |
----------------------------------------------------
states  | stname(1:n,:)   | stname((n+1):(n+nz),:) |
----------------------------------------------------
outputs | outname(cout,:) | outname(outlist,:)     |
----------------------------------------------------
```

where *cout* is the list of in 1:`rows`(p) that are not contained in outlist. (Discrete/continuous outputs may be entered in any order desired by the user.)

Example

```
octave:1> a = [1 2 3; 4 5 6; 7 8 10];
octave:2> b = [0 0 ; 0 1 ; 1 0];
octave:3> c = eye (3);
octave:4> sys = ss (a, b, c, [], 0, 3, 0, ..
>                   {"volts", "amps", "joules"});
octave:5> sysout(sys);
Input(s)
1: u_1
2: u_2

Output(s):
1: y_1
2: y_2
3: y_3

state-space form:
3 continuous states, 0 discrete states
State(s):
1: volts
2: amps
3: joules
```

```
A matrix: 3 x 3
1   2   3
4   5   6
7   8  10
B matrix: 3 x 2
0  0
0  1
1  0
C matrix: 3 x 3
1  0  0
0  1  0
0  0  1
D matrix: 3 x 3
0  0
0  0
0  0
```

Notice that the D matrix is constructed by default to the correct dimensions. Default input and output signals names were assigned since none were given

ss2sys (*a*, *b*, *c*, *d*, *tsam*, *n*, *nz*, *stname*, *inname*, *outname*, *outlist*) — Function File

Create system structure from state-space data. May be continuous, discrete, or mixed (sampled data)

Inputs

a
b
c
d usual state space matrices

default: d = zero matrix

tsam sampling rate. Default: $tsam = 0$ (continuous system)

n
nz number of continuous, discrete states in the system

If *tsam* is 0, $n =$ `rows`(a), $nz = 0$

If *tsam* is greater than zero, $n = 0$, $nz =$ `rows`(a)

see below for system partitioning

stname cell array of strings of state signal names

default (*stname*=[] on input): `x_n` for continuous states, `xd_n` for discrete states

inname cell array of strings of input signal names

default (*inname* = [] on input): `u_n`

outname cell array of strings of input signal names

default (*outname* = [] on input): `y_n`

outlist

list of indices of outputs y that are sampled

If *tsam* is 0, $outlist = []$

If *tsam* is greater than 0, $outlist = 1 : \texttt{rows}(c)$

Unlike states, discrete/continuous outputs may appear in any order

`sys2ss` returns a vector *yd* where $yd(outlist) = 1$; all other entries of *yd* are 0

Outputs $outsys$ = system data structure

System partitioning

Suppose for simplicity that outlist specified that the first several outputs were continuous and the remaining outputs were discrete. Then the system is partitioned as

```
x = [ xc ]  (n x 1)
[ xd ]  (nz x 1 discrete states)
a = [ acc acd ]  b = [ bc ]
[ adc add ]      [ bd ]
c = [ ccc ccd ]  d = [ dc ]
[ cdc cdd ]      [ dd ]

(cdc = c(outlist,1:n), etc.)
```

with dynamic equations:

$$
\begin{aligned}
\frac{d}{dt}x_c(t) &= a_{cc}x_c(t) + a_{cd}x_d(k * t_{sam}) + bc * u(t) \\
x_d((k+1) * t_{sam}) &= a_{dc}x_c(kt_{sam}) + a_{dd}x_d(kt_{sam}) + b_d u(kt_{sam}) \\
y_c(t) &= c_{cc}x_c(t) + c_{cd}x_d(kt_{sam}) + d_c u(t) \\
y_d(kt_{sam}) &= c_{dc}x_c(kt_{sam}) + c_{dd}x_d(kt_{sam}) + d_d u(kt_{sam})
\end{aligned}
$$

Signal partitions

```
| continuous      | discrete               |
----------------------------------------------------
states  | stname(1:n,:)   | stname((n+1):(n+nz),:) |
----------------------------------------------------
outputs | outname(cout,:) | outname(outlist,:)     |
----------------------------------------------------
```

where *cout* is the list of in 1:$\texttt{rows}(p)$ that are not contained in outlist. (Discrete/continuous outputs may be entered in any order desired by the user.)

Example

```
octave:1> a = [1 2 3; 4 5 6; 7 8 10];
octave:2> b = [0 0 ; 0 1 ; 1 0];
octave:3> c = eye (3);
octave:4> sys = ss (a, b, c, [], 0, 3, 0,
>                  {"volts", "amps", "joules"});
octave:5> sysout(sys);
Input(s)
```

```
1: u_1
2: u_2

Output(s):
1: y_1
2: y_2
3: y_3

state-space form:
3 continuous states, 0 discrete states
State(s):
1: volts
2: amps
3: joules

A matrix: 3 x 3
1   2   3
4   5   6
7   8  10
B matrix: 3 x 2
0  0
0  1
1  0
C matrix: 3 x 3
1  0  0
0  1  0
0  0  1
D matrix: 3 x 3
0  0
0  0
0  0
```

Notice that the *D* matrix is constructed by default to the correct dimensions. Default input and output signals names were assigned since none were given

[*a*, *b*, *c*, *d*, *tsam*, *n*, *nz*, *stname*, *inname*, *outname*, *yd*] = **sys2ss** (*sys*) — Function File

Extract state space representation from system data structure

Input

sys — System data structure

Outputs

a
b
c
d — State space matrices for *sys*

tsam — Sampling time of *sys* (0 if continuous)

n
nz Number of continuous, discrete states (discrete states come last in state vector *x*)

stname
inname
outname Signal names (lists of strings); names of states, inputs, and outputs, respectively

yd Binary vector; *yd*(*ii*) is 1 if output *y*(*ii*) is discrete (sampled); otherwise *yd*(*ii*) is 0

A warning massage is printed if the system is a mixed continuous and discrete system

Example

```
octave:1> sys=tf2sys([1 2],[3 4 5]);
octave:2> [a,b,c,d] = sys2ss(sys)
a =
0.00000   1.00000
-1.66667  -1.33333
b =
0
1
c = 0.66667  0.33333
d = 0
```

3.3 Transfer function system interface functions

tf (*num*, *den*, *tsam*, *inname*, *outname*) Function File

build system data structure from transfer function format data

Inputs

num
den coefficients of numerator/denominator polynomials

tsam sampling interval. default: 0 (continuous time)

inname
outname input/output signal names; may be a string or cell array with a single string entry

Outputs *sys* = system data structure

Example

```
octave:1> sys=tf([2 1],[1 2 1],0.1);
octave:2> sysout(sys)
Input(s)
1: u_1
Output(s):
1: y_1 (discrete)
Sampling interval: 0.1
transfer function form:
```

```
2*z^1 + 1
-----------------
1*z^2 + 2*z^1 + 1
```

tf2sys (*num*, *den*, *tsam*, *inname*, *outname*) Function File

Build system data structure from transfer function format data

Inputs

num
den Coefficients of numerator/denominator polynomials

tsam Sampling interval; default: 0 (continuous time)

inname
outname Input/output signal names; may be a string or cell array with a single string entry

Output

sys System data structure

Example

```
octave:1> sys=tf2sys([2 1],[1 2 1],0.1);
octave:2> sysout(sys)
Input(s)
1: u_1
Output(s):
1: y_1 (discrete)
Sampling interval: 0.1
transfer function form:
2*z^1 + 1
-----------------
1*z^2 + 2*z^1 + 1
```

[*num*, *den*, *tsam*, *inname*, *outname*] = **sys2tf** (*sys*) Function File

Extract transfer function data from a system data structure

See `tf` for parameter descriptions

Example

```
octave:1> sys=ss([1 -2; -1.1,-2.1],[0;1],[1 1]);
octave:2> [num,den] = sys2tf(sys)
num = 1.0000  -3.0000
den = 1.0000   1.1000  -4.3000
```

3.4 Zero-pole system interface functions

zp (*zer*, *pol*, *k*, *tsam*, *inname*, *outname*) Function File

Create system data structure from zero-pole data

Inputs

zer vector of system zeros

pol vector of system poles

k scalar leading coefficient

tsam sampling period. default: 0 (continuous system)

inname
outname input/output signal names (lists of strings)

Outputs sys: system data structure

Example

```
octave:1> sys=zp([1 -1],[-2 -2 0],1);
octave:2> sysout(sys)
Input(s)
1: u_1
Output(s):
1: y_1
zero-pole form:
1 (s - 1) (s + 1)
-----------------
s (s + 2) (s + 2)
```

zp2sys (*zer*, *pol*, *k*, *tsam*, *inname*, *outname*) Function File

Create system data structure from zero-pole data

Inputs

zer Vector of system zeros

pol Vector of system poles

k Scalar leading coefficient

tsam Sampling period; default: 0 (continuous system)

inname
outname Input/output signal names (lists of strings)

Output

sys System data structure

Example

```
octave:1> sys=zp2sys([1 -1],[-2 -2 0],1);
octave:2> sysout(sys)
Input(s)
1: u_1
Output(s):
1: y_1
zero-pole form:
1 (s - 1) (s + 1)
-----------------
s (s + 2) (s + 2)
```

[*zer*, *pol*, *k*, *tsam*, *inname*, *outname*] = **sys2zp** (*sys*) Function File

Extract zero/pole/leading coefficient information from a system data structure

See `zp` for parameter descriptions

Example

```
octave:1> sys=ss([1 -2; -1.1,-2.1],[0;1],[1 1]);
octave:2> [zer,pol,k] = sys2zp(sys)
zer = 3.0000
pol =
-2.6953
1.5953
k = 1
```

3.5 Data structure access functions

syschnames (*sys*, *opt*, *list*, *names*) Function File

Superseded by `syssetsignals`

syschtsam (*sys*, *tsam*) Function File

This function changes the sampling time (tsam) of the system. Exits with an error if sys is purely continuous time

[*n*, *nz*, *m*, *p*, *yd*] = **sysdimensions** (*sys*, *opt*) Function File

return the number of states, inputs, and/or outputs in the system *sys*

Inputs

sys system data structure

opt String indicating which dimensions are desired. Values:

- `"all"` (default) return all parameters as specified under Outputs below
- `"cst"` return *n*= number of continuous states
- `"dst"` return *n*= number of discrete states
- `"in"` return *n*= number of inputs
- `"out"` return *n*= number of outputs

Outputs

n number of continuous states (or individual requested dimension as specified by *opt*)

nz number of discrete states

m number of system inputs

p number of system outputs

yd binary vector; $yd(ii)$ is nonzero if output *ii* is discrete $yd(ii) = 0$ if output *ii* is continuous

See also: sysgetsignals, sysgettsam

[*stname*, *inname*, *outname*, *yd*] = **sysgetsignals** (*sys*) Function File
siglist = **sysgetsignals** (*sys*, *sigid*) Function File
signame = **sysgetsignals** (*sys*, *sigid*, *signum*, *strflg*) Function File

Get signal names from a system

Inputs

sys system data structure for the state space system

sigid signal id. String. Must be one of

- `"in"` input signals
- `"out"` output signals
- `"st"` stage signals
- `"yd"` value of logical vector *yd*

signum index(indices) or name(s) or signals; see `sysidx`

strflg flag to return a string instead of a cell array; Values:

- `0` (default) return a cell array (even if signum specifies an individual signal)
- `1` return a string. Exits with an error if signum does not specify an individual signal

Outputs

•If *sigid* is not specified:

stname
inname
outname signal names (cell array of strings); names of states, inputs, and outputs, respectively

yd binary vector; *yd*(*ii*) is nonzero if output *ii* is discrete

•If *sigid* is specified but *signum* is not specified:

`sigid="in"`
siglist is set to the cell array of input names

`sigid="out"`
siglist is set to the cell array of output names

`sigid="st"`
siglist is set to the cell array of state names
stage signals

`sigid="yd"`
siglist is set to logical vector indicating discrete outputs; *siglist*(*ii*) $= 0$ indicates that output *ii* is continuous (unsampled), otherwise it is discrete

•If the first three input arguments are specified:
signame is a cell array of the specified signal names (*sigid* is `"in"`, `"out"`, or `"st"`), or else the logical flag indicating whether output(s) *signum* is(are) discrete (*sigval*=1) or continuous (*sigval*=0)

Examples (From `sysrepdemo`)

```
octave> sys=ss(rand(4),rand(4,2),rand(3,4));
octave># get all signal names
octave> [Ast,Ain,Aout,Ayd] = sysgetsignals(sys)
Ast =
(
[1] = x_1
[2] = x_2
[3] = x_3
[4] = x_4
)
Ain =
(
[1] = u_1
[2] = u_2
)
Aout =
(
[1] = y_1
[2] = y_2
[3] = y_3
)
Ayd =

0 0 0
octave> # get only input signal names:
octave> Ain = sysgetsignals(sys,"in")
Ain =
(
[1] = u_1
[2] = u_2
)
octave> # get name of output 2 (in cell array):
octave> Aout = sysgetsignals(sys,"out",2)
Aout =
(
[1] = y_2
)
octave> # get name of output 2 (as string):
octave> Aout = sysgetsignals(sys,"out",2,1)
Aout = y_2
```

sysgettype (*sys*) Function File

return the initial system type of the system

Input

sys System data structure

Output

systype String indicating how the structure was initially constructed. Values: `"ss"`, `"zp"`, or `"tf"`

FIR initialized systems return `systype="tf"`

syssetsignals (*sys*, *opt*, *names*, *sig_idx*) Function File

change the names of selected inputs, outputs and states

Inputs

sys System data structure

opt Change default name (output)

- `"out"` Change selected output names
- `"in"` Change selected input names
- `"st"` Change selected state names
- `"yd"` Change selected outputs from discrete to continuous or from continuous to discrete

names

- `opt = "out", "in", "st"`
  string or string array containing desired signal names or values
- `opt = "yd"`
  To desired output continuous/discrete flag Set name to 0 for continuous, or 1 for discrete

sig_idx indices or names of outputs, yd, inputs, or states whose respective names/values should be changed

Default: replace entire cell array of names/entire yd vector

Outputs

retsys *sys* with appropriate signal names changed (or *yd* values, where appropriate)

Example

```
octave:1> sys=ss ([1 2; 3 4],[5;6],[7 8]);
octave:2> sys = syssetsignals (sys, "st",
>                             str2mat("Posx","Velx"));
octave:3> sysout(sys)
Input(s)
1: u_1
Output(s):
1: y_1
state-space form:
2 continuous states, 0 discrete states
State(s):
1: Posx
```

```
2: Velx
A matrix: 2 x 2
1  2
3  4
B matrix: 2 x 1
5
6
C matrix: 1 x 2
7  8
D matrix: 1 x 1
0
```

sysupdate (*sys*, *opt*) Function File

Update the internal representation of a system

Inputs

sys: system data structure

opt string:

- `"tf"` update transfer function form
- `"zp"` update zero-pole form
- `"ss"` update state space form
- `"all"` all of the above

Outputs

retsys Contains union of data in sys and requested data If requested data in *sys* is already up to date then *retsys*=*sys*

Conversion to `tf` or `zp` exits with an error if the system is mixed continuous/digital

See also: tf, ss, zp, sysout, sys2ss, sys2tf, sys2zp

[*systype*, *nout*, *nin*, *ncstates*, *ndstates*] = **minfo** (*inmat*) Function File

Determines the type of system matrix. *inmat* can be a varying, a system, a constant, and an empty matrix

Outputs

systype Can be one of: varying, system, constant, and empty

nout The number of outputs of the system

nin The number of inputs of the system

ncstates The number of continuous states of the system

ndstates The number of discrete states of the system

sysgettsam (*sys*) Function File

Return the sampling time of the system *sys*

4 System display functions

sysout (*sys*, *opt*) Function File

print out a system data structure in desired format

sys system data structure

opt Display option

`[]` primary system form (default)

`"ss"` state space form

`"tf"` transfer function form

`"zp"` zero-pole form

`"all"` all of the above

tfout (*num*, *denom*, *x*) Function File

Print formatted transfer function $n(s)/d(s)$ to the screen *x* defaults to the string `"s"` See also: polyval, polyvalm, poly, roots, conv, deconv, residue, filter, polyderiv, polyinteg, polyout

zpout (*zer*, *pol*, *k*, *x*) Function File

print formatted zero-pole form to the screen *x* defaults to the string `"s"` See also: polyval, polyvalm, poly, roots, conv, deconv, residue, filter, polyderiv, polyinteg, polyout

5 Block Diagram Manipulations

See Chapter 8 [systime], page 37.

Unless otherwise noted, all parameters (input,output) are system data structures.

bddemo (*inputs*) Function File

Octave Controls toolbox demo: Block Diagram Manipulations demo

buildssic (*clst*, *ulst*, *olst*, *ilst*, *s1*, *s2*, *s3*, *s4*, *s5*, *s6*, *s7*, *s8*) Function File

Form an arbitrary complex (open or closed loop) system in state-space form from several systems. `buildssic` can easily (despite its cryptic syntax) integrate transfer functions from a complex block diagram into a single system with one call This function is especially useful for building open loop interconnections for $\mathcal{H}_\infty$ and $\mathcal{H}_2$ designs or for closing loops with these controllers

Although this function is general purpose, the use of `sysgroup` `sysmult`, `sysconnect` and the like is recommended for standard operations since they can handle mixed discrete and continuous systems and also the names of inputs, outputs, and states

The parameters consist of 4 lists that describe the connections outputs and inputs and up to 8 systems *s1*–*s8* Format of the lists:

clst connection list, describes the input signal of each system. The maximum number of rows of Clst is equal to the sum of all inputs of s1-s8

Example: `[1 2 -1; 2 1 0]` means that: new input 1 is old input 1 + output 2 - output 1, and new input 2 is old input 2 + output 1. The order of rows is arbitrary

ulst if not empty the old inputs in vector *ulst* will be appended to the outputs. You need this if you want to "pull out" the input of a system. Elements are input numbers of *s1*–*s8*

olst output list, specifies the outputs of the resulting systems. Elements are output numbers of *s1*–*s8* The numbers are allowed to be negative and may appear in any order. An empty matrix means all outputs

ilst input list, specifies the inputs of the resulting systems. Elements are input numbers of *s1*–*s8* The numbers are allowed to be negative and may appear in any order. An empty matrix means all inputs

Example: Very simple closed loop system

```
w        e  +-----+   u  +-----+
--->o--*-->|  K  |--*-->|  G  |--*---> y
^  |   +-----+  |   +-----+  |
- |  |           |           |
|  |          +---------------> u
|  |                         |
|  +-------------------------|---> e
|                            |
+----------------------------+
```

The closed loop system GW can be obtained by

```
GW = buildssic([1 2; 2 -1], 2, [1 2 3], 2, G, K);
```

clst 1st row: connect input 1 (G) with output 2 (K)
2nd row: connect input 2 (K) with negative output 1 (G)

ulst Append input of 2 (K) to the number of outputs

olst Outputs are output of 1 (G), 2 (K) and appended output 3 (from *ulst*)

ilst The only input is 2 (K)

Here is a real example:

```
+----+
-------------------->| W1 |---> v1
z   |                     +----+
----|-------------+
|                 |
|    +---+    v       +----+
*--->| G |--->O--*-->| W2 |---> v2
|    +---+       |    +----+
|                |
|                v
u                 y
```

$$\min\|GW_{vz}\|_\infty$$

The closed loop system GW from $[z, u]^T$ to $[v_1, v_2, y]^T$ can be obtained by (all SISO systems):

```
GW = buildssic([1, 4; 2, 4; 3, 1], 3, [2, 3, 5],
[3, 4], G, W1, W2, One);
```

where "One" is a unity gain (auxiliary) function with order 0 (e.g. `One = ugain(1);`)

sys = **jet707** () Function File

Creates a linearized state-space model of a Boeing 707-321 aircraft at v=80 m/s ($M = 0.26$, $G_{a0} = -3°$, $\alpha_0 = 4°$, $\kappa = 50°$)

System inputs: (1) thrust and (2) elevator angle

System outputs: (1) airspeed and (2) pitch angle

Reference: R. Brockhaus: *Flugregelung* (Flight Control), Springer, 1994 See also: ord2

ord2 (*nfreq*, *damp*, *gain*) Function File

Creates a continuous 2nd order system with parameters:

Inputs

nfreq natural frequency [Hz]. (not in rad/s)

damp damping coefficient

gain dc-gain This is steady state value only for damp > 0 gain is assumed to be 1.0 if omitted

Output

outsys system data structure has representation with $w = 2\pi f$:

```
/                                              \
| / -2w*damp -w \  / w \                       |
G = | |              |, |   |, [ 0  gain ], 0 |
| \   w       0 /  \ 0 /                       |
\                                              /
```

See also `jet707` (MIMO example, Boeing 707-321 aircraft model)

sysadd (*gsys*, *hsys*) Function File

returns *sys* = *gsys* + *hsys*

- Exits with an error if *gsys* and *hsys* are not compatibly dimensioned
- Prints a warning message is system states have identical names; duplicate names are given a suffix to make them unique
- *sys* input/output names are taken from *gsys*

```
--------
----|  gsys  |---
u   |    ----------  +|
-----                (_)----> y
|     ________   +|
----|  hsys  |---
--------
```

sys = **sysappend** (*syst*, *b*, *c*, *d*, *outname*, *inname*, *yd*) Function File

appends new inputs and/or outputs to a system

Inputs

syst system data structure

b matrix to be appended to sys "B" matrix (empty if none)

c matrix to be appended to sys "C" matrix (empty if none)

d revised sys d matrix (can be passed as [] if the revised d is all zeros)

outname list of names for new outputs

inname list of names for new inputs

yd binary vector; $yd(ii) = 0$ indicates a continuous output; $yd(ii) = 1$ indicates a discrete output

Outputs

sys

```
sys.b := [syst.b , b]
sys.c := [syst.c  ]
[ c     ]
sys.d := [syst.d | D12 ]
[ D21   | D22 ]
```

where $D12$, $D21$, and $D22$ are the appropriate dimensioned blocks of the input parameter *d*

- The leading block $D11$ of d is ignored
- If *inname* and *outname* are not given as arguments, the new inputs and outputs are be assigned default names
- *yd* is a binary vector of length rows(c) that indicates continuous/sampled outputs. Default value for *yd* is:
  - *sys* is continuous or mixed *yd* = `zeros(1,rows(c))`
  - *sys* is discrete *yd* = `ones(1,rows(c))`

clsys = **sysconnect** (*sys*, *out_idx*, *in_idx*, *order*, *tol*) Function File

Close the loop from specified outputs to respective specified inputs

Inputs

sys System data structure

out_idx
in_idx Names or indices of signals to connect (see `sysidx`) The output specified by *out_idx*(*ii*) is connected to the input specified by *in_idx*(*ii*)

order logical flag (default = 0)

`0` Leave inputs and outputs in their original order

`1` Permute inputs and outputs to the order shown in the diagram below

tol Tolerance for singularities in algebraic loops, default: 200`eps`

Outputs

clsys Resulting closed loop system

Method

`sysconnect` internally permutes selected inputs, outputs as shown below, closes the loop, and then permutes inputs and outputs back to their original order

```
--------------------
u_1       ----->|                   |----> y_1
|        sys       |
old u_2 |                   |
u_2* ---->(+)--->|                   |----->y_2
(in_idx)   ^     --------------------    | (out_idx)
|                              |
-------------------------------
```

The input that has the summing junction added to it has an * added to the end of the input name

[*csys*, *acd*, *ccd*] = **syscont** (*sys*) Function File

Extract the purely continuous subsystem of an input system

Input

sys system data structure

Outputs

csys is the purely continuous input/output connections of *sys*

acd
ccd connections from discrete states to continuous states, discrete states to continuous outputs, respectively

If no continuous path exists, *csys* will be empty

[*dsys*, *adc*, *cdc*] = **sysdisc** (*sys*) Function File

Input

sys System data structure

Outputs

dsys Purely discrete portion of sys (returned empty if there is no purely discrete path from inputs to outputs)

adc
cdc Connections from continuous states to discrete states and discrete outputs, respectively

retsys = **sysdup** (*asys*, *out_idx*, *in_idx*) Function File

Duplicate specified input/output connections of a system

Inputs

asys system data structure

out_idx
in_idx indices or names of desired signals (see `sigidx`) duplicates are made of `y(out_idx(ii))` and `u(in_idx(ii))`

Output

retsys Resulting closed loop system: duplicated i/o names are appended with a `"+"` suffix

Method

`sysdup` creates copies of selected inputs and outputs as shown below. *u1*, *y1* is the set of original inputs/outputs, and *u2*, *y2* is the set of duplicated inputs/outputs in the order specified in *in_idx*, *out_idx*, respectively

```
--------------------
u1  ----->|                  |----> y1
|       asys       |
u2 ------>|                  |----->y2
(in_idx)  -------------------- (out_idx)
```

sys = **sysgroup** (*asys*, *bsys*) Function File

Combines two systems into a single system

Inputs

asys
bsys System data structures

Output

sys $sys = \mathrm{blockdiag}(asys, bsys)$

```
-------------------
|    --------     |
u1 ----->|--> | asys |--->|----> y1
|    --------     |
|    --------     |
u2 ----->|--> | bsys |--->|----> y2
|    --------     |
-------------------
Ksys
```

The function also rearranges the internal state-space realization of *sys* so that the continuous states come first and the discrete states come last If there are duplicate names, the second name has a unique suffix appended on to the end of the name

sys = **sysmult** (*Asys*, *Bsys*) Function File

Compute $sys = Asys * Bsys$ (series connection):

```
u   ----------      ----------
--->|  Bsys  |---->|  Asys  |--->
----------      ----------
```

A warning occurs if there is direct feed-through from an input or a continuous state of *Bsys*, through a discrete output of *Bsys*, to a continuous state or output in *Asys* (system data structure does not recognize discrete inputs)

retsys = **sysprune** (*asys*, *out_idx*, *in_idx*) Function File

Extract specified inputs/outputs from a system

Inputs

asys system data structure

out_idx
in_idx Indices or signal names of the outputs and inputs to be kept in the returned system; remaining connections are "pruned" off May select as [] (empty matrix) to specify all outputs/inputs

```
retsys = sysprune (Asys, [1:3,4], "u_1");
retsys = sysprune (Asys, {"tx", "ty", "tz"}, 4);
```

Output

retsys Resulting system

```
--------------------
u1 ------->|                  |----> y1
(in_idx)  |       Asys       | (out_idx)
u2 ------->|                  |----| y2
(deleted)-------------------- (deleted)
```

pv = **sysreorder** (*vlen*, *list*) Function File

Inputs

vlen Vector length

list A subset of `[1:vlen]`

Output

pv A permutation vector to order elements of `[1:vlen]` in `list` to the end of a vector

Used internally by `sysconnect` to permute vector elements to their desired locations

retsys = **sysscale** (*sys*, *outscale*, *inscale*, *outname*, *inname*) Function File

scale inputs/outputs of a system

Inputs

sys Structured system

outscale
inscale Constant matrices of appropriate dimension

outname
inname Lists of strings with the names of respectively outputs and inputs

Output

retsys resulting open loop system:

```
-----------    -------    -----------
u --->| inscale |--->| sys |--->| outscale |---> y
-----------    -------    -----------
```

If the input names and output names (each a list of strings) are not given and the scaling matrices are not square, then default names will be given to the inputs and/or outputs

A warning message is printed if outscale attempts to add continuous system outputs to discrete system outputs; otherwise *yd* is set appropriately in the returned value of *sys*

sys = **syssub** (*Gsys*, *Hsys*) Function File

Return $sys = Gsys - Hsys$

Method

Gsys and *Hsys* are connected in parallel The input vector is connected to both systems; the outputs are subtracted. Returned system names are those of *Gsys*

```
     +--------+
+--->|  Gsys  |---+
|    +--------+   |
|                +|
u --+                (_)--> y
|                -|
|    +--------+   |
+--->|  Hsys  |---+
     +--------+
```

ugain (*n*) Function File

Creates a system with unity gain, no states This trivial system is sometimes needed to create arbitrary complex systems from simple systems with `buildssic` Watch out if you are forming sampled systems since `ugain` does not contain a sampling period See also: hinfdemo, jet707

W = **wgt1o** (*vl*, *vh*, *fc*) Function File

State space description of a first order weighting function

Weighting function are needed by the $\mathcal{H}_2/\mathcal{H}_\infty$ design procedure These functions are part of the augmented plant P (see `hinfdemo` for an application example)

Inputs

vl Gain at low frequencies

vh Gain at high frequencies

fc Corner frequency (in Hz, **not** in rad/sec)

Output

W Weighting function, given in form of a system data structure

ksys = **parallel** (*asys*, *bsys*) Function File

Forms the parallel connection of two systems

```
--------------------
|      --------    |
u  ----->|----> | asys |--->|----> y1
|    |      --------    |
|    |      --------    |
|--->|----> | bsys |--->|----> y2
|      --------    |
--------------------
ksys
```

[*retsys*, *nc*, *no*] = **sysmin** (*sys*, *flg*) Function File

Returns a minimal (or reduced order) system

Inputs

sys System data structure

flg When equal to 0 (default value), returns minimal system, in which state names are lost; when equal to 1, returns system with physical states removed that are either uncontrollable or unobservable (cannot reduce further without discarding physical meaning of states)

Outputs

retsys Returned system

nc Number of controllable states in the returned system

no Number of observable states in the returned system

cflg `is_controllable(retsys)`

oflg `is_observable(retsys)`

6 Numerical Functions

x = **are** (*a*, *b*, *c*, *opt*) Function File

Solve the Algebraic Riccati Equation

$$A^T X + XA - XBX + C = 0$$

Inputs for identically dimensioned square matrices

a — n by n matrix;

b — n by n matrix or n by m matrix; in the latter case b is replaced by $b := b * b'$;

c — n by n matrix or p by m matrix; in the latter case c is replaced by $c := c' * c$;

opt — (optional argument; default = `"B"`): String option passed to `balance` prior to ordered Schur decomposition

Output

x — solution of the ARE

Method Laub's Schur method (IEEE Transactions on Automatic Control, 1979) is applied to the appropriate Hamiltonian matrix See also: balance, dare

x = **dare** (*a*, *b*, *q*, *r*, *opt*) Function File

Return the solution, *x* of the discrete-time algebraic Riccati equation

$$A^T XA - X + A^T XB(R + B^T XB)^{-1} B^T XA + Q = 0$$

Inputs

a — n by n matrix;

b — n by m matrix;

q — n by n matrix, symmetric positive semidefinite, or a p by n matrix, In the latter case $q := q' * q$ is used;

r — m by m, symmetric positive definite (invertible);

opt — (optional argument; default = `"B"`): String option passed to `balance` prior to ordered QZ decomposition

Output

x — solution of DARE

Method Generalized eigenvalue approach (Van Dooren; SIAM J Sci. Stat. Comput., Vol 2) applied to the appropriate symplectic pencil

See also: Ran and Rodman, *Stable Hermitian Solutions of Discrete Algebraic Riccati Equations*, Mathematics of Control, Signals and Systems, Vol 5, no 2 (1992), pp 165–194 See also: balance, are

[*tvals*, *plist*] = **dre** (*sys*, *q*, *r*, *qf*, *t0*, *tf*, *ptol*, *maxits*) Function File

Solve the differential Riccati equation

$$-\frac{dP}{dt} = A^T P + PA - PBR^{-1}B^T P + Q$$

$$P(t_f) = Q_f$$

for the LTI system sys. Solution of standard LTI state feedback optimization

$$\min \int_{t_0}^{t_f} x^T Qx + u^T Rudt + x(t_f)^T Q_f x(t_f)$$

optimal input is

$$u = -R^{-1}B^T P(t)x$$

Inputs

sys continuous time system data structure

q state integral penalty

r input integral penalty

qf state terminal penalty

t0
tf limits on the integral

ptol tolerance (used to select time samples; see below); default = 0.1

maxits number of refinement iterations (default=10)

Outputs

tvals time values at which $p(t)$ is computed

plist list values of $p(t)$; *plist* { *i* } is $p(tvals(i))$

tvals is selected so that:

$$\|plist_i - plist_{i-1}\| < ptol$$

for every i between 2 and length(*tvals*)

dgram (*a*, *b*) Function File

Return controllability gramian of discrete time system

$$x_{k+1} = ax_k + bu_k$$

Inputs

a n by n matrix

b n by m matrix

Output

m — *n* by *n* matrix, satisfies

$$ama^T - m + bb^T = 0$$

dlyap (*a*, *b*) — Function File

Solve the discrete-time Lyapunov equation

Inputs

a — *n* by *n* matrix;

b — Matrix: *n* by *n*, *n* by *m*, or *p* by *n*

Output

x — matrix satisfying appropriate discrete time Lyapunov equation

Options:

- *b* is square: solve
$$axa^T - x + b = 0$$
- *b* is not square: *x* satisfies either
$$axa^T - x + bb^T = 0$$
or
$$a^T xa - x + b^T b = 0,$$
whichever is appropriate

Method Uses Schur decomposition method as in Kitagawa, *An Algorithm for Solving the Matrix Equation* $X = FXF' + S$, International Journal of Control, Volume 25, Number 5, pages 745–753 (1977)

Column-by-column solution method as suggested in Hammarling, *Numerical Solution of the Stable, Non-Negative Definite Lyapunov Equation*, IMA Journal of Numerical Analysis, Volume 2, pages 303–323 (1982)

W = **gram** (*sys*, *mode*) — Function File
Wc = **gram** (*a*, *b*) — Function File

`gram (`*sys*`, 'c')` returns the controllability gramian of the (continuous- or discrete-time) system *sys* `gram (`*sys*`, 'o')` returns the observability gramian of the (continuous- or discrete-time) system *sys* `gram (`*a*`, `*b*`)` returns the controllability gramian *Wc* of the continuous-time system $dx/dt = ax+bu$; i.e., *Wc* satisfies $aWc+mWc'+bb' = 0$

lyap (*a*, *b*, *c*) — Function File
lyap (*a*, *b*) — Function File

Solve the Lyapunov (or Sylvester) equation via the Bartels-Stewart algorithm (Communications of the ACM, 1972)

If *a*, *b*, and *c* are specified, then `lyap` returns the solution of the Sylvester equation

$$AX + XB + C = 0$$

If only `(a, b)` are specified, then `lyap` returns the solution of the Lyapunov equation

$$A^T X + XA + B = 0$$

If *b* is not square, then `lyap` returns the solution of either

$$A^T X + XA + B^T B = 0$$

or

$$AX + XA^T + BB^T = 0$$

whichever is appropriate

Solves by using the Bartels-Stewart algorithm (1972)

qzval (*a*, *b*) Function File

Compute generalized eigenvalues of the matrix pencil $(A - \lambda B)$

a and *b* must be real matrices

`qzval` is obsolete; use `qz` instead

y = **zgfmul** (*a*, *b*, *c*, *d*, *x*) Function File

Compute product of *zgep* incidence matrix F with vector *x* Used by `zgepbal` (in `zgscal`) as part of generalized conjugate gradient iteration

zgfslv (*n*, *m*, *p*, *b*) Function File

Solve system of equations for dense zgep problem

zz = **zginit** (*a*, *b*, *c*, *d*) Function File

Construct right hand side vector *zz* for the zero-computation generalized eigenvalue problem balancing procedure. Called by `zgepbal`

zgreduce (*sys*, *meps*) Function File

Implementation of procedure REDUCE in (Emami-Naeini and Van Dooren, Automatica, # 1982)

[*nonz*, *zer*] = **zgrownorm** (*mat*, *meps*) Function File

Return *nonz* = number of rows of *mat* whose two norm exceeds *meps*, and *zer* = number of rows of mat whose two norm is less than *meps*

x = **zgscal** (*f*, *z*, *n*, *m*, *p*) Function File

Generalized conjugate gradient iteration to solve zero-computation generalized eigenvalue problem balancing equation $fx = z$; called by `zgepbal`

[a, b] = **zgsgiv** (*c*, *s*, *a*, *b*) Function File

Apply givens rotation c,s to row vectors *a*, *b* No longer used in zero-balancing (__zgpbal__); kept for backward compatibility

x = **zgshsr** (*y*) Function File

Apply householder vector based on e^m to column vector *y* Called by `zgfslv`

References

ZGEP Hodel, *Computation of Zeros with Balancing*, 1992, Linear Algebra and its Applications

Generalized CG
Golub and Van Loan, *Matrix Computations, 2nd ed* 1989.

7 System Analysis-Properties

analdemo () — Function File

Octave Controls toolbox demo: State Space analysis demo

[*n*, *m*, *p*] = **abcddim** (*a*, *b*, *c*, *d*) — Function File

Check for compatibility of the dimensions of the matrices defining the linear system $[A, B, C, D]$ corresponding to

$$\frac{dx}{dt} = Ax + Bu$$
$$y = Cx + Du$$

or a similar discrete-time system

If the matrices are compatibly dimensioned, then `abcddim` returns

n The number of system states

m The number of system inputs

p The number of system outputs

Otherwise `abcddim` returns $n = m = p = -1$

Note: n = 0 (pure gain block) is returned without warning See also: is_abcd

ctrb (*sys*, *b*) — Function File
ctrb (*a*, *b*) — Function File

Build controllability matrix:

$$Q_s = [BABA^2B \ldots A^{n-1}B]$$

of a system data structure or the pair (*a*, *b*)

`ctrb` forms the controllability matrix The numerical properties of `is_controllable` are much better for controllability tests

h2norm (*sys*) — Function File

Computes the $\mathcal{H}_2$ norm of a system data structure (continuous time only)

Reference: Doyle, Glover, Khargonekar, Francis, *State-Space Solutions to Standard $\mathcal{H}_2$ and $\mathcal{H}_\infty$ Control Problems*, IEEE TAC August 1989

[*g*, *gmin*, *gmax*] = **hinfnorm** (*sys*, *tol*, *gmin*, *gmax*, *ptol*) — Function File

Computes the $\mathcal{H}_\infty$ norm of a system data structure

Inputs

sys system data structure

tol $\mathcal{H}_\infty$ norm search tolerance (default: 0.001)

gmin minimum value for norm search (default: 1e-9)

gmax maximum value for norm search (default: 1e+9)

ptol pole tolerance:

- if sys is continuous, poles with $|\text{real}(pole)| < ptol\|H\|$ (H is appropriate Hamiltonian) are considered to be on the imaginary axis
- if sys is discrete, poles with $|\text{pole} - 1| < ptol\|[s_1 s_2]\|$ (appropriate symplectic pencil) are considered to be on the unit circle
- Default value: 1e-9

Outputs

g Computed gain, within *tol* of actual gain. *g* is returned as Inf if the system is unstable

gmin
gmax Actual system gain lies in the interval [*gmin*, *gmax*]

References: Doyle, Glover, Khargonekar, Francis, *State-space solutions to standard $\mathcal{H}_2$ and $\mathcal{H}_\infty$ control problems*, IEEE TAC August 1989; Iglesias and Glover, *State-Space approach to discrete-time $\mathcal{H}_\infty$ control*, Int. J. Control, vol 54, no. 5, 1991; Zhou, Doyle, Glover, *Robust and Optimal Control*, Prentice-Hall, 1996

obsv (*sys*, *c*) Function File
obsv (*a*, *c*) Function File

Build observability matrix:

$$Q_b = \begin{bmatrix} C \\ CA \\ CA^2 \\ \vdots \\ CA^{n-1} \end{bmatrix}$$

of a system data structure or the pair (*a*, *c*)

The numerical properties of `is_observable` are much better for observability tests

[*zer*, *pol*] = **pzmap** (*sys*) Function File

Plots the zeros and poles of a system in the complex plane

Input

sys System data structure

Outputs

pol

zer if omitted, the poles and zeros are plotted on the screen otherwise, *pol* and *zer* are returned as the system poles and zeros (see `sys2zp` for a preferable function call)

retval = **is_abcd** (*a*, *b*, *c*, *d*) Function File

Returns *retval* = 1 if the dimensions of *a*, *b*, *c*, *d* are compatible, otherwise *retval* = 0 with an appropriate diagnostic message printed to the screen. The matrices *b*, *c*, or *d* may be omitted See also: abcddim

[*retval*, *u*] = **is_controllable** (*sys*, *tol*) Function File
[*retval*, *u*] = **is_controllable** (*a*, *b*, *tol*) Function File

Logical check for system controllability

Inputs

sys system data structure

a
b *n* by *n*, *n* by *m* matrices, respectively

tol optional roundoff parameter. Default value: `10*eps`

Outputs

retval Logical flag; returns true (1) if the system *sys* or the pair (*a*, *b*) is controllable, whichever was passed as input arguments

u *u* is an orthogonal basis of the controllable subspace

Method Controllability is determined by applying Arnoldi iteration with complete re-orthogonalization to obtain an orthogonal basis of the Krylov subspace

```
span ([b,a*b,...,a^{n-1}*b])
```

The Arnoldi iteration is executed with `krylov` if the system has a single input; otherwise a block Arnoldi iteration is performed with `krylovb` See also: size, rows, columns, length, ismatrix, isscalar, isvector, is_observable, is_stabilizable, is_detectable, krylov, krylovb

retval = **is_detectable** (*a*, *c*, *tol*, *dflg*) Function File
retval = **is_detectable** (*sys*, *tol*) Function File

Test for detectability (observability of unstable modes) of (*a*, *c*)

Returns 1 if the system *a* or the pair (*a*, *c*) is detectable, 0 if not, and -1 if the system has unobservable modes at the imaginary axis (unit circle for discrete-time systems)

See `is_stabilizable` for detailed description of arguments and computational method See also: is_stabilizable, size, rows, columns, length, ismatrix, isscalar, isvector

[*retval*, *dgkf_struct*] = **is_dgkf** (*asys*, *nu*, *ny*, *tol*) Function File

Determine whether a continuous time state space system meets assumptions of DGKF algorithm Partitions system into:

```
[dx/dt]    [A  | Bw  Bu  ][w]
[ z   ] = [Cz | Dzw Dzu ][u]
[ y   ]    [Cy | Dyw Dyu ]
```

or similar discrete-time system If necessary, orthogonal transformations *qw*, *qz* and nonsingular transformations *ru*, *ry* are applied to respective vectors *w*, *z*, *u*, *y* in order to satisfy DGKF assumptions Loop shifting is used if *dyu* block is nonzero

Inputs

asys system data structure

nu number of controlled inputs

ny number of measured outputs

tol threshold for 0; default: 200*`eps`

Outputs

retval true(1) if system passes check, false(0) otherwise

dgkf_struct
data structure of `is_dgkf` results. Entries:

nw
nz dimensions of *w*, *z*

a system A matrix

bw (n x nw) qw-transformed disturbance input matrix

bu (n x nu) ru-transformed controlled input matrix;
$B = [BwBu]$

cz (nz x n) Qz-transformed error output matrix

cy (ny x n) ry-transformed measured output matrix
$C = [Cz; Cy]$

dzu

dyw off-diagonal blocks of transformed system D matrix that enter z, y from u, w respectively

ru controlled input transformation matrix

ry observed output transformation matrix

dyu_nz nonzero if the *dyu* block is nonzero

dyu untransformed *dyu* block

dflg nonzero if the system is discrete-time

`is_dgkf` exits with an error if the system is mixed discrete/continuous

References

[1] Doyle, Glover, Khargonekar, Francis, *State Space Solutions to Standard $\mathcal{H}_2$ and $\mathcal{H}_\infty$ Control Problems*, IEEE TAC August 1989

[2] Maciejowksi, J.M., *Multivariable Feedback Design*, Addison-Wesley, 1989

digital = **is_digital** (*sys*, *eflg*) Function File

Return nonzero if system is digital

Inputs

sys System data structure

eflg When equal to 0 (default value), exits with an error if the system is mixed (continuous and discrete components); when equal to 1, print a warning if the system is mixed (continuous and discrete); when equal to 2, operate silently

Output

digital When equal to 0, the system is purely continuous; when equal to 1, the system is purely discrete; when equal to -1, the system is mixed continuous and discrete

Exits with an error if *sys* is a mixed (continuous and discrete) system

[*retval*, *u*] = **is_observable** (*a*, *c*, *tol*) Function File
[*retval*, *u*] = **is_observable** (*sys*, *tol*) Function File

Logical check for system observability

Default: tol = `tol = 10*norm(a,'fro')*eps`

Returns 1 if the system *sys* or the pair (*a*, *c*) is observable, 0 if not

See `is_controllable` for detailed description of arguments and default values See also: size, rows, columns, length, ismatrix, isscalar, isvector

is_sample (*ts*) Function File

Return true if *ts* is a valid sampling time (real, scalar, > 0)

is_siso (*sys*) Function File

Returns nonzero if the system data structure *sys* is single-input, single-output

retval = **is_stabilizable** (*sys*, *tol*) Function File
retval = **is_stabilizable** (*a*, *b*, *tol*, *dflg*) Function File

Logical check for system stabilizability (i.e., all unstable modes are controllable) Returns 1 if the system is stabilizable, 0 if the system is not stabilizable, -1 if the system has non stabilizable modes at the imaginary axis (unit circle for discrete-time systems

Test for stabilizability is performed via Hautus Lemma. If *dflg*$\neq$0 assume that discrete-time matrices (a,b) are supplied See also: size, rows, columns, length, ismatrix, isscalar, isvector, is_observable, is_stabilizable, is_detectable

is_signal_list (*mylist*) Function File

Return true if *mylist* is a list of individual strings

is_stable (*a*, *tol*, *dflg*) Function File
is_stable (*sys*, *tol*) Function File

Returns 1 if the matrix *a* or the system *sys* is stable, or 0 if not

Inputs

tol is a roundoff parameter, set to 200*`eps` if omitted

dflg Digital system flag (not required for system data structure):

dflg `!= 0` stable if eig(a) is in the unit circle

dflg `== 0` stable if eig(a) is in the open LHP (default)

See also: size, rows, columns, length, ismatrix, isscalar, isvector, is_observable, is_stabilizable, is_detectable, krylov, krylovb

8 System Analysis-Time Domain

c2d (*sys*, *opt*, *t*) Function File
c2d (*sys*, *t*) Function File

Converts the system data structure describing:

$$\dot{x} = A_c x + B_c u$$

into a discrete time equivalent model:

$$x_{n+1} = A_d x_n + B_d u_n$$

via the matrix exponential or bilinear transform

Inputs

sys system data structure (may have both continuous time and discrete time subsystems)

opt string argument; conversion option (optional argument; may be omitted as shown above)

- `"ex"` use the matrix exponential (default)
- `"bi"` use the bilinear transformation

$$s = \frac{2(z-1)}{T(z+1)}$$

FIXME: This option exits with an error if *sys* is not purely continuous. (The `ex` option can handle mixed systems.)

- `"matched"`
  Use the matched pole/zero equivalent transformation (currently only works for purely continuous SISO systems)

t sampling time; required if *sys* is purely continuous

Note that if the second argument is not a string, `c2d()` assumes that the second argument is t and performs appropriate argument checks

Output

dsys Discrete time equivalent via zero-order hold, sample each t sec

This function adds the suffix `_d` to the names of the new discrete states

d2c (*sys*, *tol*) Function File
d2c (*sys*, *opt*) Function File

Convert a discrete (sub)system into a purely continuous one The sampling time used is `sysgettsam(`*sys*`)`

Inputs

sys system data structure with discrete components

tol Scalar value Tolerance for convergence of default `"log"` option (see below)

opt conversion option. Choose from:

`"log"` (default) Conversion is performed via a matrix logarithm Due to some problems with this computation, it is followed by a steepest descent algorithm to identify continuous time *a*, *b*, to get a better fit to the original data

If called as `d2c (`*sys*`, `*tol*`)`, with *tol* positive scalar, the `"log"` option is used. The default value for *tol* is `1e-8`

`"bi"` Conversion is performed via bilinear transform $z = (1+sT/2)/(1-sT/2)$ where T is the system sampling time (see `sysgettsam`)

FIXME: bilinear option exits with an error if *sys* is not purely discrete

Output

csys continuous time system (same dimensions and signal names as in *sys*)

[*dsys*, *fidx*] = **dmr2d** (*sys*, *idx*, *sprefix*, *ts2*, *cuflg*) Function File

convert a multirate digital system to a single rate digital system states specified by *idx*, *sprefix* are sampled at *ts2*, all others are assumed sampled at *ts1* = `sysgettsam (`*sys*`)`

Inputs

sys discrete time system; `dmr2d` exits with an error if *sys* is not discrete

idx indices or names of states with sampling time `sysgettsam(`*sys*`)` (may be empty); see `cellidx`

sprefix list of string prefixes of states with sampling time `sysgettsam(`*sys*`)` (may be empty)

ts2 sampling time of states not specified by *idx*, *sprefix* must be an integer multiple of `sysgettsam(`*sys*`)`

cuflg "constant u flag" if *cuflg* is nonzero then the system inputs are assumed to be constant over the revised sampling interval *ts2* Otherwise, since the inputs can change during the interval t in $[kts2, (k+1)ts2]$, an additional set of inputs is included in the revised B matrix so that these intersample inputs may be included in the single-rate system default *cuflg* = 1

Outputs

dsys equivalent discrete time system with sampling time *ts2*

The sampling time of sys is updated to *ts2*

if *cuflg*=0 then a set of additional inputs is added to the system with suffixes _d1, ..., _dn to indicate their delay from the starting time k *ts2*, i.e u = [u_1; u_1_d1; ..., u_1_dn] where u_1_dk is the input k*ts1 units of time after u_1 is sampled. (*ts1* is the original sampling time of the discrete time system and *ts2* = (n+1)*ts1)

fidx indices of "formerly fast" states specified by *idx* and *sprefix*; these states are updated to the new (slower) sampling interval *ts2*

WARNING Not thoroughly tested yet; especially when *cuflg* == 0

damp (*p*, *tsam*) Function File

Displays eigenvalues, natural frequencies and damping ratios of the eigenvalues of a matrix *p* or the *A* matrix of a system *p*, respectively If *p* is a system, *tsam* must not be specified If *p* is a matrix and *tsam* is specified, eigenvalues of *p* are assumed to be in *z*-domain See also: eig

dcgain (*sys*, *tol*) Function File

Returns dc-gain matrix. If dc-gain is infinite an empty matrix is returned The argument *tol* is an optional tolerance for the condition number of the *A* Matrix in *sys* (default *tol* = 1.0e-10)

[*y*, *t*] = **impulse** (*sys*, *inp*, *tstop*, *n*) Function File

Impulse response for a linear system The system can be discrete or multivariable (or both) If no output arguments are specified, `impulse` produces a plot or the impulse response data for system *sys*

Inputs

sys System data structure

inp Index of input being excited

tstop The argument *tstop* (scalar value) denotes the time when the simulation should end

n the number of data values

Both parameters *tstop* and *n* can be omitted and will be computed from the eigenvalues of the A Matrix

Outputs

y Values of the impulse response

t Times of the impulse response

See also: step

[*y*, *t*] = **step** (*sys*, *inp*, *tstop*, *n*) Function File

Step response for a linear system The system can be discrete or multivariable (or both) If no output arguments are specified, `step` produces a plot or the step response data for system *sys*

Inputs

sys System data structure

inp Index of input being excited

tstop The argument *tstop* (scalar value) denotes the time when the simulation should end

n the number of data values

Both parameters *tstop* and n can be omitted and will be computed from the eigenvalues of the A Matrix

Outputs

y Values of the step response

t Times of the step response

When invoked with the output parameter y the plot is not displayed See also: impulse

9 System Analysis-Frequency Domain

Demonstration/tutorial script

frdemo () — Function File

Octave Control Toolbox demo: Frequency Response demo

[*mag*, *phase*, *w*] = **bode** (*sys*, *w*, *out_idx*, *in_idx*) — Function File

If no output arguments are given: produce Bode plots of a system; otherwise, compute the frequency response of a system data structure

Inputs

sys — a system data structure (must be either purely continuous or discrete; see is_digital)

w — frequency values for evaluation

if *sys* is continuous, then bode evaluates $G(jw)$ where $G(s)$ is the system transfer function

if *sys* is discrete, then bode evaluates G(`exp`(jwT)), where

- T is the system sampling time
- $G(z)$ is the system transfer function

Default the default frequency range is selected as follows: (These steps are **not** performed if *w* is specified)

1. via routine __bodquist__, isolate all poles and zeros away from w=0 (jw=0 or `exp`(jwT)=1) and select the frequency range based on the breakpoint locations of the frequencies
2. if *sys* is discrete time, the frequency range is limited to jwT in $[0, 2\pi/T]$
3. A "smoothing" routine is used to ensure that the plot phase does not change excessively from point to point and that singular points (e.g., crossovers from +/- 180) are accurately shown

out_idx
in_idx — The names or indices of outputs and inputs to be used in the frequency response. See `sysprune`

Example

```
bode(sys,[],"y_3", {"u_1","u_4"});
```

Outputs

mag
phase — the magnitude and phase of the frequency response $G(jw)$ or $G(\texttt{exp}(jwT))$ at the selected frequency values

w — the vector of frequency values used

1. If no output arguments are given, e.g.,

```
bode(sys);
```

bode plots the results to the screen. Descriptive labels are automatically placed

Failure to include a concluding semicolon will yield some garbage being printed to the screen (`ans = []`)

2. If the requested plot is for an MIMO system, mag is set to $||G(jw)||$ or $||G(\mathtt{exp}(jwT))||$ and phase information is not computed

[*wmin*, *wmax*] = **bode_bounds** (*zer*, *pol*, *dflg*, *tsam*) — Function File

Get default range of frequencies based on cutoff frequencies of system poles and zeros Frequency range is the interval $[10^{w_{min}}, 10^{w_{max}}]$

Used internally in `__freqresp__` (`bode`, `nyquist`)

freqchkw (*w*) — Function File

Used by `__freqresp__` to check that input frequency vector *w* is valid Returns boolean value

out = **ltifr** (*a*, *b*, *w*) — Function File
out = **ltifr** (*sys*, *w*) — Function File

Linear time invariant frequency response of single-input systems

Inputs

a
b — coefficient matrices of $dx/dt = Ax+Bu$

sys — system data structure

w — vector of frequencies

Output

out — frequency response, that is:

$$G(j\omega) = (j\omega I - A)^{-1}B$$

for complex frequencies $s = jw$

[*realp*, *imagp*, *w*] = **nyquist** (*sys*, *w*, *out_idx*, *in_idx*, *atol*) — Function File
nyquist (*sys*, *w*, *out_idx*, *in_idx*, *atol*) — Function File

Produce Nyquist plots of a system; if no output arguments are given, Nyquist plot is printed to the screen

Compute the frequency response of a system

Inputs (pass as empty to get default values)

sys — system data structure (must be either purely continuous or discrete; see `is_digital`)

w — frequency values for evaluation If sys is continuous, then bode evaluates $G(jw)$; if sys is discrete, then bode evaluates $G(exp(jwT))$, where T is the system sampling time

default the default frequency range is selected as follows: (These steps are **not** performed if *w* is specified)

1. via routine `__bodquist__`, isolate all poles and zeros away from w=0 (jw=0 or $exp(jwT) = 1$) and select the frequency range based on the breakpoint locations of the frequencies
2. if *sys* is discrete time, the frequency range is limited to jwT in $[0, 2p\pi]$
3. A "smoothing" routine is used to ensure that the plot phase does not change excessively from point to point and that singular points (e.g., crossovers from +/- 180) are accurately shown

atol for interactive nyquist plots: atol is a change-in-slope tolerance for the of asymptotes (default = 0; 1e-2 is a good choice). This allows the user to "zoom in" on portions of the Nyquist plot too small to be seen with large asymptotes

Outputs

realp
imagp the real and imaginary parts of the frequency response $G(jw)$ or $G(exp(jwT))$ at the selected frequency values

w the vector of frequency values used

If no output arguments are given, nyquist plots the results to the screen If *atol* != 0 and asymptotes are detected then the user is asked interactively if they wish to zoom in (remove asymptotes) Descriptive labels are automatically placed

Note: if the requested plot is for an MIMO system, a warning message is presented; the returned information is of the magnitude $\|G(jw)\|$ or $\|G(\exp(jwT)\|$ only; phase information is not computed

[*mag*, *phase*, *w*] = **nichols** (*sys*, *w*, *outputs*, *inputs*) Function File

Produce Nichols plot of a system

Inputs

sys System data structure (must be either purely continuous or discrete; see `is_digital`)

w Frequency values for evaluation

if sys is continuous, then nichols evaluates $G(jw)$

if sys is discrete, then nichols evaluates $G(exp(jwT))$, where T=*sys. tsam* is the system sampling time

the default frequency range is selected as follows (These steps are **not** performed if *w* is specified):

1. via routine `__bodquist__`, isolate all poles and zeros away from w=0 ($jw = 0$ or $exp(jwT) = 1$) and select the frequency range based on the breakpoint locations of the frequencies
2. if sys is discrete time, the frequency range is limited to jwT in $[0, 2p\pi]$

3. A "smoothing" routine is used to ensure that the plot phase does not change excessively from point to point and that singular points (e.g., crossovers from +/- 180) are accurately shown

outputs
inputs the names or indices of the output(s) and input(s) to be used in the frequency response; see `sysprune`

Outputs

mag
phase The magnitude and phase of the frequency response $G(jw)$ or $G(exp(jwT))$ at the selected frequency values

w The vector of frequency values used

If no output arguments are given, `nichols` plots the results to the screen Descriptive labels are automatically placed. See `xlabel`, `ylabel`, and `title`

Note: if the requested plot is for an MIMO system, *mag* is set to $\|G(jw)\|$ or $\|G(\exp(jwT)\|$ and phase information is not computed

[*zer*, *gain*] = **tzero** (*a*, *b*, *c*, *d*, *opt*) Function File
[*zer*, *gain*] = **tzero** (*sys*, *opt*) Function File

Compute transmission zeros of a continuous system:

$$\dot{x} = Ax + Bu$$

$$y = Cx + Du$$

or of a discrete one:

$$x_{k+1} = Ax_k + Bu_k$$

$$y_k = Cx_k + Du_k$$

Outputs

zer transmission zeros of the system

gain leading coefficient (pole-zero form) of SISO transfer function returns gain=0 if system is multivariable

References

1. Emami-Naeini and Van Dooren, Automatica, 1982
2. Hodel, *Computation of Zeros with Balancing*, 1992 Lin. Alg. Appl

zr = **tzero2** (*a*, *b*, *c*, *d*, *bal*) Function File

Compute the transmission zeros of *a*, *b*, *c*, *d*

bal = balancing option (see balance); default is `"B"`

Needs to incorporate `mvzero` algorithm to isolate finite zeros; use `tzero` instead

10 Controller Design

dgkfdemo () Function File

Octave Controls toolbox demo: $\mathcal{H}_2/\mathcal{H}_\infty$ options demos

hinfdemo () Function File

$\mathcal{H}_\infty$ design demos for continuous SISO and MIMO systems and a discrete system. The SISO system is difficult to control because it is non-minimum-phase and unstable. The second design example controls the `jet707` plant, the linearized state space model of a Boeing 707-321 aircraft at v=80 m/s ($M = 0.26$, $G_{a0} = -3°$, $\alpha_0 = 4°$, $\kappa = 50°$) Inputs: (1) thrust and (2) elevator angle Outputs: (1) airspeed and (2) pitch angle. The discrete system is a stable and second order

SISO plant:

$$G(s) = \frac{s-2}{(s+2)(s-1)}$$

```
+-----+
-------------------->| W1 |---> v1
z   |                    +-----+
----|-------------+
|             |
|    +---+    v   y  +-----+
u *--->| G |--->O--*-->| W2 |---> v2
|    +---+       |   +-----+
|                |
|    +---+       |
-----| K |<-------
+---+
```

$$\min \|T_{vz}\|_\infty$$

W1 und *W2* are the robustness and performance weighting functions

MIMO plant:

The optimal controller minimizes the $\mathcal{H}_\infty$ norm of the augmented plant P (mixed-sensitivity problem):

```
w
1 -----------+
|                   +----+
+---------------------->| W1 |----> z1
w         |   |                    +----+
2 ------------------------+
|   |            |
|   v   +----+   v       +----+
+--*-->o-->| G  |-->o--*-->| W2 |---> z2
|          +----+      |   +----+
|                      |
^                      v
u                       y (to K)
(from controller K)
```

$$\begin{bmatrix} z_1 \\ z_2 \\ y \end{bmatrix} = P \begin{bmatrix} w_1 \\ w_2 \\ u \end{bmatrix}$$

Discrete system:

This is not a true discrete design. The design is carried out in continuous time while the effect of sampling is described by a bilinear transformation of the sampled system This method works quite well if the sampling period is "small" compared to the plant time constants

The continuous plant:

$$G(s) = \frac{1}{(s+2)(s+1)}$$

is discretised with a ZOH (Sampling period = $Ts = 1$ second):

$$G(z) = \frac{0.199788z + 0.073498}{(z-0.36788)(z-0.13534)}$$

```
+----+
-------------------->| W1 |---> v1
z   |                      +----+
----|-------------+
|             |
|    +---+    v       +----+
*--->| G |--->O--*-->| W2 |---> v2
|    +---+       |    +----+
|                |
|    +---+       |
-----| K |<-------
+---+
```

$$\min \|T_{vz}\|_\infty$$

W1 and *W2* are the robustness and performance weighting functions

[*l*, *m*, *p*, *e*] = **dlqe** (*a*, *g*, *c*, *sigw*, *sigv*, *z*) Function File

Construct the linear quadratic estimator (Kalman filter) for the discrete time system

$$x_{k+1} = Ax_k + Bu_k + Gw_k$$

$$y_k = Cx_k + Du_k + v_k$$

where *w*, *v* are zero-mean gaussian noise processes with respective intensities *sigw* = `cov (`*w*`, `*w*`)` and *sigv* = `cov (`*v*`, `*v*`)`

If specified, *z* is `cov (`*w*`, `*v*`)`. Otherwise `cov (`*w*`, `*v*`) = 0`

The observer structure is

$$z_{k|k} = z_{k|k-1} + l(y_k - Cz_{k|k-1} - Du_k)$$

$$z_{k+1|k} = Az_{k|k} + Bu_k$$

The following values are returned:

l The observer gain, $(A - ALC)$ is stable

m The Riccati equation solution

p The estimate error covariance after the measurement update

e The closed loop poles of $(A - ALC)$

[*k*, *p*, *e*] = **dlqr** (*a*, *b*, *q*, *r*, *z*) Function File

Construct the linear quadratic regulator for the discrete time system

$$x_{k+1} = Ax_k + Bu_k$$

to minimize the cost functional

$$J = \sum x^T Qx + u^T Ru$$

z omitted or

$$J = \sum x^T Qx + u^T Ru + 2x^T Zu$$

z included

The following values are returned:

k The state feedback gain, $(A - BK)$ is stable

p The solution of algebraic Riccati equation

e The closed loop poles of $(A - BK)$

[*Lp*, *Lf*, *P*, *Z*] = **dkalman** (*A*, *G*, *C*, *Qw*, *Rv*, *S*) Function File

Construct the linear quadratic estimator (Kalman predictor) for the discrete time system

$$x_{k+1} = Ax_k + Bu_k + Gw_k$$

$$y_k = Cx_k + Du_k + v_k$$

where *w*, *v* are zero-mean gaussian noise processes with respective intensities *Qw* = `cov (`*w*`, `*w*`)` and *Rv* = `cov (`*v*`, `*v*`)`

If specified, S is `cov (`*w*`, `*v*`)`. Otherwise `cov (`*w*`, `*v*`) = 0`

The observer structure is $x_{k+1|k} = Ax_{k|k-1} + Bu_k + L_p(y_k - Cx_{k|k-1} - Du_k)$ $x_{k|k} = x_{k|k} + L_f(y_k - Cx_{k|k-1} - Du_k)$

The following values are returned:

Lp The predictor gain, $(A - L_pC)$ is stable

Lf The filter gain

P The Riccati solution $P = E\{(x - x_{n|n-1})(x - x_{n|n-1})'\}$

Z The updated error covariance matrix $Z = E\{(x - x_{n|n})(x - x_{n|n})'\}$

[*K*, *gain*, *kc*, *kf*, *pc*, *pf*] = **h2syn** (*asys*, *nu*, *ny*, *tol*) Function File

Design $\mathcal{H}_2$ optimal controller per procedure in Doyle, Glover, Khargonekar, Francis, *State-Space Solutions to Standard $\mathcal{H}_2$ and $\mathcal{H}_\infty$ Control Problems*, IEEE TAC August 1989

Discrete-time control per Zhou, Doyle, and Glover, *Robust and optimal control*, Prentice-Hall, 1996

Inputs

asys system data structure (see ss, sys2ss)
- controller is implemented for continuous time systems
- controller is **not** implemented for discrete time systems

nu number of controlled inputs

ny number of measured outputs

tol threshold for 0. Default: 200*`eps`

Outputs

k system controller

gain optimal closed loop gain

kc full information control (packed)

kf state estimator (packed)

pc ARE solution matrix for regulator subproblem

pf ARE solution matrix for filter subproblem

K = **hinf_ctr** (*dgs*, *f*, *h*, *z*, *g*) Function File

Called by `hinfsyn` to compute the $\mathcal{H}_\infty$ optimal controller

Inputs

dgs data structure returned by `is_dgkf`

f
h feedback and filter gain (not partitioned)

g final gamma value

Outputs

K controller (system data structure)

Do not attempt to use this at home; no argument checking performed

[*k*, *g*, *gw*, *xinf*, *yinf*] = **hinfsyn** (*asys*, *nu*, *ny*, *gmin*, *gmax*, *gtol*, *ptol*, *tol*) Function File

Inputs input system is passed as either

asys system data structure (see `ss`, `sys2ss`)

- controller is implemented for continuous time systems
- controller is **not** implemented for discrete time systems (see bilinear transforms in `c2d`, `d2c`)

nu number of controlled inputs

ny number of measured outputs

gmin initial lower bound on $\mathcal{H}_\infty$ optimal gain

gmax initial upper bound on $\mathcal{H}_\infty$ Optimal gain

gtol Gain threshold. Routine quits when *gmax*/*gmin* < 1+tol

ptol poles with `abs(real(pole))` $< ptol\|H\|$ (H is appropriate Hamiltonian) are considered to be on the imaginary axis Default: 1e-9

tol threshold for 0. Default: 200*`eps`

gmax, *min*, *tol*, and *tol* must all be positive scalars

Outputs

k System controller

g Designed gain value

gw Closed loop system

xinf ARE solution matrix for regulator subproblem

yinf ARE solution matrix for filter subproblem

References:

1. Doyle, Glover, Khargonekar, Francis, *State-Space Solutions to Standard $\mathcal{H}_2$ and $\mathcal{H}_\infty$ Control Problems*, IEEE TAC August 1989

2. Maciejowksi, J.M., *Multivariable feedback design*, Addison-Wesley, 1989, ISBN 0-201-18243-2
3. Keith Glover and John C. Doyle, *State-space formulae for all stabilizing controllers that satisfy an $\mathcal{H}_\infty$ norm bound and relations to risk sensitivity*, Systems & Control Letters 11, Oct. 1988, pp 167–172

[*retval*, *pc*, *pf*] = **hinfsyn_chk** (*a*, *b1*, *b2*, *c1*, *c2*, *d12*, *d21*, *g*, *ptol*) — Function File

Called by `hinfsyn` to see if gain *g* satisfies conditions in Theorem 3 of Doyle, Glover, Khargonekar, Francis, *State Space Solutions to Standard $\mathcal{H}_2$ and $\mathcal{H}_\infty$ Control Problems*, IEEE TAC August 1989

Warning: do not attempt to use this at home; no argument checking performed

Inputs

As returned by `is_dgkf`, except for:

g — candidate gain level

ptol — as in `hinfsyn`

Outputs

retval — 1 if g exceeds optimal Hinf closed loop gain, else 0

pc — solution of "regulator" $\mathcal{H}_\infty$ ARE

pf — solution of "filter" $\mathcal{H}_\infty$ ARE

Do not attempt to use this at home; no argument checking performed

[*xinf*, *x_ha_err*] = **hinfsyn_ric** (*a*, *bb*, *c1*, *d1dot*, *r*, *ptol*) — Function File

Forms

```
xx = ([bb; -c1'*d1dot]/r) * [d1dot'*c1 bb'];
Ha = [a 0*a; -c1'*c1 - a'] - xx;
```

and solves associated Riccati equation The error code *x_ha_err* indicates one of the following conditions:

0 — successful

1 — *xinf* has imaginary eigenvalues

2 — *hx* not Hamiltonian

3 — *xinf* has infinite eigenvalues (numerical overflow)

4 — *xinf* not symmetric

5 — *xinf* not positive definite

6 — *r* is singular

[*k*, *p*, *e*] = **lqe** (*a*, *g*, *c*, *sigw*, *sigv*, *z*) Function File

Construct the linear quadratic estimator (Kalman filter) for the continuous time system

$$\frac{dx}{dt} = Ax + Gu$$
$$y = Cx + v$$

where *w* and *v* are zero-mean gaussian noise processes with respective intensities

```
sigw = cov (w, w)
sigv = cov (v, v)
```

The optional argument *z* is the cross-covariance `cov (w, v)`. If it is omitted, `cov (w, v) = 0` is assumed

Observer structure is `dz/dt = A z + B u + k (y - C z - D u)`

The following values are returned:

k The observer gain, $(A - KC)$ is stable

p The solution of algebraic Riccati equation

e The vector of closed loop poles of $(A - KC)$

[*k*, *q1*, *p1*, *ee*, *er*] = **lqg** (*sys*, *sigw*, *sigv*, *q*, *r*, *in_idx*) Function File

Design a linear-quadratic-gaussian optimal controller for the system

```
dx/dt = A x + B u + G w       [w]=N(0,[Sigw 0    ])
y = C x + v                 [v]  (    0   Sigv ])
```

or

```
x(k+1) = A x(k) + B u(k) + G w(k)   [w]=N(0,[Sigw 0    ])
y(k) = C x(k) + v(k)              [v]  (    0   Sigv ])
```

Inputs

sys system data structure

sigw
sigv intensities of independent Gaussian noise processes (as above)

q
r state, control weighting respectively. Control ARE is

in_idx names or indices of controlled inputs (see `sysidx`, `cellidx`)
default: last dim(R) inputs are assumed to be controlled inputs, all others are assumed to be noise inputs

Outputs

k system data structure format LQG optimal controller (Obtain A, B, C matrices with `sys2ss`, `sys2tf`, or `sys2zp` as appropriate)

p1 Solution of control (state feedback) algebraic Riccati equation

q1 Solution of estimation algebraic Riccati equation

ee Estimator poles

es Controller poles

See also: h2syn, lqe, lqr

[k, p, e] = **lqr** (a, b, q, r, z) Function File

construct the linear quadratic regulator for the continuous time system

$$\frac{dx}{dt} = Ax + Bu$$

to minimize the cost functional

$$J = \int_0^\infty x^T Q x + u^T R u$$

z omitted or

$$J = \int_0^\infty x^T Q x + u^T R u + 2x^T Z u$$

z included

The following values are returned:

k The state feedback gain, $(A - BK)$ is stable and minimizes the cost functional

p The stabilizing solution of appropriate algebraic Riccati equation

e The vector of the closed loop poles of $(A - BK)$

Reference Anderson and Moore, *Optimal control: linear quadratic methods*, Prentice-Hall, 1990, pp. 56–58

[y, x] = **lsim** (*sys*, u, t, *x0*) Function File

Produce output for a linear simulation of a system; produces a plot for the output of the system, *sys*

u is an array that contains the system's inputs. Each row in u corresponds to a different time step. Each column in u corresponds to a different input. t is an array that contains the time index of the system; t should be regularly spaced. If initial conditions are required on the system, the *x0* vector should be added to the argument list

When the lsim function is invoked a plot is not displayed; however, the data is returned in y (system output) and x (system states)

K = **place** (*sys*, p) Function File
K = **place** (a, b, p) Function File

Computes the matrix K such that if the state is feedback with gain K, then the eigenvalues of the closed loop system (i.e. $A - BK$) are those specified in the vector p

Version: Beta (May-1997): If you have any comments, please let me know (see the file place.m for my address)

11 Miscellaneous Functions (Not yet properly filed/documented)

axis2dlim (*axdata*) Function File

Determine axis limits for 2-D data (column vectors); leaves a 10% margin around the plots Inserts margins of +/- 0.1 if data is one-dimensional (or a single point)

Input

axdata n by 2 matrix of data $[x, y]$

Output

axvec Vector of axis limits appropriate for call to `axis` function

moddemo (*inputs*) Function File

Octave Control toolbox demo: Model Manipulations demo

prompt (*str*) Function File

Prompt user to continue

Input

str Input string. Its default value is:

```
\n ---- Press a key to  continue ---
```

rldemo (*inputs*) Function File

Octave Control toolbox demo: Root Locus demo

[*rldata*, *k*] = **rlocus** (*sys*[, *increment*, *min_k*, *max_k*]) Function File

Display root locus plot of the specified SISO system

```
-----   ---     --------
--->| + |---|k|---->| SISO |----------->
-----   ---     --------        |
- ^                             |
|_____________________________|
```

Inputs

sys system data structure

min_k Minimum value of k

max_k Maximum value of k

increment The increment used in computing gain values

Outputs

Plots the root locus to the screen

rldata Data points plotted: in column 1 real values, in column 2 the imaginary values

k Gains for real axis break points

[*yy*, *idx*] = **sortcom** (*xx*[, *opt*]) Function File

Sort a complex vector

Inputs

xx Complex vector

opt sorting option:

- `"re"` Real part (default);
- `"mag"` By magnitude;
- `"im"` By imaginary part

if *opt* is not chosen as `"im"`, then complex conjugate pairs are grouped together, $a - jb$ followed by $a+jb$

Outputs

yy Sorted values

idx Permutation vector: `yy = xx(idx)`

[*num*, *den*] = **ss2tf** (*a*, *b*, *c*, *d*) Function File

Conversion from transfer function to state-space The state space system:

$$\dot{x} = Ax + Bu$$

$$y = Cx + Du$$

is converted to a transfer function:

$$G(s) = \frac{\text{num}(s)}{\text{den}(s)}$$

used internally in system data structure format manipulations

[*pol*, *zer*, *k*] = **ss2zp** (*a*, *b*, *c*, *d*) Function File

Converts a state space representation to a set of poles and zeros; *k* is a gain associated with the zeros

Used internally in system data structure format manipulations

starp (*P*, *K*, *ny*, *nu*) Function File

Redheffer star product or upper/lower LFT, respectively

```
+-------+
--------->|       |--------->
|   P   |
+--->|       |---+  ny
|    +-------+   |
+-------------------+
|  |
+----------------+  |
|                   |
|    +-------+      |
+--->|       |------+ nu
|   K   |
--------->|       |--------->
+-------+
```

If *ny* and *nu* “consume” all inputs and outputs of K then the result is a lower fractional transformation If *ny* and *nu* “consume” all inputs and outputs of P then the result is an upper fractional transformation

ny and/or *nu* may be negative (i.e. negative feedback)

[*a*, *b*, *c*, *d*] = **tf2ss** (*num*, *den*) — Function File

Conversion from transfer function to state-space The state space system:

$$\dot{x} = Ax + Bu$$
$$y = Cx + Du$$

is obtained from a transfer function:

$$G(s) = \frac{\text{num}(s)}{\text{den}(s)}$$

The vector *den* must contain only one row, whereas the vector *num* may contain as many rows as there are outputs y of the system. The state space system matrices obtained from this function will be in controllable canonical form as described in *Modern Control Theory*, (Brogan, 1991)

[*zer*, *pol*, *k*] = **tf2zp** (*num*, *den*) — Function File

Converts transfer functions to poles-and-zero representations

Returns the zeros and poles of the SISO system defined by *num*/*den* *k* is a gain associated with the system zeros

[*a*, *b*, *c*, *d*] = **zp2ss** (*zer*, *pol*, *k*) — Function File

Conversion from zero / pole to state space

Inputs

zer
pol — Vectors of (possibly) complex poles and zeros of a transfer function. Complex values must come in conjugate pairs (i.e., $x+jy$ in *zer* means that $x - jy$ is also in *zer*) The number of zeros must not exceed the number of poles

k Real scalar (leading coefficient)

Outputs

a
b
c
d The state space system, in the form:

$$\dot{x} = Ax + Bu$$

$$y = Cx + Du$$

[*num*, *den*] = **zp2tf** (*zer*, *pol*, *k*) Function File

Converts zeros / poles to a transfer function

Inputs

zer
pol Vectors of (possibly complex) poles and zeros of a transfer function. Complex values must appear in conjugate pairs

k Real scalar (leading coefficient)
